# Supplementary material for: Spatiotemporal mosaic self-patterning of pluripotent stem cells using CRISPR interference
Source: eLife. 2018 Oct 9;7:e36045. doi: 10.7554/eLife.36045 (PMC6177255; doi:10.7554/eLife.36045)
Supplement: Supplementary file 1. — (1) Table 1 displays guide RNA sequences used for CRISPRi knockdown. (2) Table 2 outlines primer sequences used for gene expression analysis by quantitative PCR. (3) Table 3 shows gene targets and primer sequences used for the Fluidigm 96.96 array gene expression analysis. [file elife-36045-supp1.docx]

**Supplementary Table 1**

| Guide RNA (gRNA) Target | Location to TSS | Sequence |
| --- | --- | --- |
| CDH1 | -141 | TCACCGCGTCTATGCGAGGC |
| CDH1 | -161 | CACCCGGCCTCGCATAGACG |
| CDH1 | -68 | CCCGTACCGCTGATTGGCTG |
| CDH1 | -46 | TCAGCCAATCAGCGGTACGG |
| CDH1 | -6 | GCAGTTCCGACGCCACTGAG |
| ROCK1 (Mandegar et al., 2016) | +11 | CGGGGCGCGGACGCTCGGAA |
| KCNH2 (Off Target Guide) | -175 | TTCTGGGCGCGCGAGTCCCA |

**Supplementary Table 2**

| Gene Description | Gene Symbol | Forward Primer | Reverse Primer |
| --- | --- | --- | --- |
| 18S ribosomal RNA | 18S | CTTCCACAGGAGGCCTACAC | CTTCGGCCCACACCCTTAAT |
| Rho associated coiled-coil containing protein kinase 1 | ROCK1 | GTTCCCCTTCCGAGCGTC | TGTCCGCCTTCCTGTTCAAA |
| E-cadherin | CDH1 | CGGGAATGCAGTTGAGGATC | AGGATGGTGTAAGCGATGGC |
| POU domain, class 5, transcription factor 1 | POU5F1 | ATGCATTCAAACTGAGGTGCCT | AACTTCACCTTCCCTCCAACCA |
| SRY box 2 | SOX2 | TCAGGAGTTGTCAAGGCAGAG | GCCGCCGCCGATGATTTGTTAT |
| Homeobox protein nanog | NANOG | CAATGGTGTGACGCAGGGAT | TGCACCAGGTCTGAGTGTTC |
| Brachyury | BRA (T) | TTTCCAGATGGTGAGAGCCG | CCGATGCCTCAACTCTCCAG |
| SRY box 9 | SOX9 | GAGCGAGCGGTGCATTTG | TGGTGTTCTGAGAGGCACAG |

**Supplementary Table 3**

| Gene Name | Gene Symbol | Forward Primer | Reverse Primer |
| --- | --- | --- | --- |
| 18S ribosomal RNA | 18S | CTTCCACAGGAGGCCTACAC | CTTCGGCCCACACCCTTAAT |
| Polyubiquitin C precursor | UBC | AGTAGTCCCTTCTCGGCGAT | GACGATCACAGCGATCCACA |
| POU domain, class 5, transcription factor 1 | POU5F1 | ATGCATTCAAACTGAGGTGCCT | AACTTCACCTTCCCTCCAACCA |
| SRY box 2 | SOX2 | TTTGTCGGAGACGGAGAAGC | TAACTGTCCATGCGCTGGTT |
| Homeobox protein nanog | NANOG | CAATGGTGTGACGCAGGGAT | TGCACCAGGTCTGAGTGTTC |
| C-myc protein | MYC | CAAGAGGCGAACACACAACG | GTCGTTTCCGCAACAAGTCC |
| Fibroblast growth factor 8 | FGF8 | GCGCATCCCTAGTGAAGGAG | CCGTCTCCACGATGAGCTTT |
| Fibroblast growth factor 4 | FGF4 | AGTACCCCGGCATGTTCATC | TCATCCGAAGAAAGTGCACCA |
| Fibroblast growth factor receptor 1 | FGFR1 | GTCTGCTGACTCCAGTGCAT | ACGGTTGGGTTTGTCCTTGT |
| Fibroblast growth factor receptor 2 | FGFR2 | ACAGTTTCGGCTGAGTCCAG | CATGACCACTTGCCCAAAGC |
| Fibroblast growth factor receptor 3 | FGFR3 | AGGAGCTCTTCAAGCTGCTG | ACAGGTCCAGGTACTCGTCG |
| Fibroblast growth factor receptor 4 | FGFR4 | GAGGAGGACCCCACATGGA | TACTACCTGGCCAAAGCAGC |
| Epidermal growth factor receptor | EGFR | CTAAGATCCCGTCCATCGCC | GGAGCCCAGCACTTTGATCT |
| Epidermal growth factor | EGF | GTCTTGACTCTACTCCACCCC | CTCGGTACTGACATCGCTCC |
| Nodal | NODAL | CTGGAGGTGCTGCTTTCAGG | CCCATCCACTGCCACATCTT |
| Bone morphogenic protein 4 | BMP4 | CGGAAGCTAGGTGAGTGTGG | CATAGGTCCCTGCAGTAGCG |
| Bone morphogenic protein 4 | BMP7 | CACTCGAGCTTCATCCACCG | GGGTACTGAAGACGGCCTTG |
| Cerberus 1 | CER1 | TTTGCTTTGGGAAATGCGGG | CTCCGTCTTCACCTTGCACT |
| Wnt family, member 2 | WNT2 | GCTACGACACCTCCCATGTC | GGTCATGTAGCGGTTGTCCA |
| Wnt family, member 3 | WNT3 | CACAACACGAGGACGGAGAA | GCTTCCCATGAGACTTCGCT |
| Wnt family, member 5A | WNT5A | TCTGGCTCCACTTGTTGCTC | CGACCACCAAGAATTGGCTTC |
| Wnt family, member 11 | WNT11 | TCTTTGGGGTGGCACTTCTC | TGCCGAGTTCACTTGACGAG |
| Inhibin A | INHBA | GCTCAGACAGCTCTTACCACA | CCTCTCAGCCAAAGCAAGGG |
| Transforming growth factor beta 1 | TGFB1 | CTGTCCAACATGATCGTGCG | TGACACAGAGATCCGCAGTC |
| Transforming growth factor beta 2 | TGFB2 | CGACGAAGAGTACTACGCCA | TCAAGGTACCCACAGAGCAC |
| SMAD family, member 1 | SMAD1 | TGTATTCGTGAGTTCGCGGT | CCAAATGCAAAAGGACAGCAGA |
| SMAD family, member 2 | SMAD2 | GCTCCCTCCGTCTTCCATAC | CTTGTATCGAACCTCCCGGC |
| Dickkopf-related protein 1 | DKK1 | GGGTCTTTGTCGCGATGGTA | CTGGTACTTATTCCCGCCCG |
| Dickkopf-related protein 2 | DKK2 | GGTACTCGGCACAGAGATCG | CCCTGATGGAGCACTGGTTT |
| Left right determination factor 1 | LEFTY1 | GCCATCGAGGGACTTGACTT | AAACTGAGCAAGGGCTCTCC |
| CAMP responsive element binding protein 1 | CREB1 | CTCAGCCGGGTACTACCATTC | CATGTTACCATCTTCAAACTGACG |
| Fos proto-oncogene | FOS | CCGAGCTGGTGCATTACAGA | ACACACTCCATGCGTTTTGC |
| Jun proto-oncogene | JUN | GTGCCGAAAAAGGAAGCTGG | CTGCGTTAGCATGAGTTGGC |
| Notch 1 | NOTCH1 | GCAAGAACGCCGGGACA | GGCTGGCACGATTTCCCTGA |
| Notch 2 | NOTCH2 | GATACAGATGCGAGTGTGTCCC | AGACAATGCCCTGGATGGAAAA |
| Notch 3 | NOTCH3 | GGACGTCAGTGTGAACTCCT | GAAACTCCCTGCCAGGTTGG |
| Notch 4 | NOTCH4 | GAGACGTGCCAGTTTCCTGA | GAGGCAAGTGCACAAGAAGC |
| GATA binding protein 4 | GATA4 | ACCTGGGACTTGGAGGATAGCAAA | CCATCAGCGTGTAAAGGCATCTGA |
| Delta like canonical notch ligand 1 | DLL1 | GGAGGCACTGTGACGACAA | GCACACTCGCACACATAGC |
| Chordin | CHRD | TATGCCTTGGACGAGACGTG | GGTTGGGCACTCTGGTTTGA |
| Caudal type homeobox 2 | CDX2 | GCAGCCAAGTGAAAACCAGG | TTCCTCTCCTTTGCTCTGCG |
| Actin, alpha 2 | ACTA2 | AAAGCAAGTCCTCCAGCGTT | TAGTCCCGGGGATAGGCAAA |
| GATA binding protein 6 | GATA6 | TCTCCATGTGCATTGGGGAC | AAGGAAATCGCCCTGTTCGT |
| SRY-box 17 | SOX17 | GGACCGCACGGAATTTGAAC | TAATATACCGCGGAGCTGGC |
| Eomesdoermin | EOMES | AGTCACCTTCTTCCAGCGTG | CCTCTTCCGAGGGGAAGGTA |
| Alpha fetoprotein | AFP | CTGCTGCAGCCAAAGTGAAG | ATAGCGAGCAGCCCAAAGAA |
| Hes family BHLH transcription factor 1 | HES1 | AAAAATTCCTCGTCCCCGGT | ATGCCGCGAGCTATCTTTCT |
| Brachyury transcription factor | T | TTTCCAGATGGTGAGAGCCG | CCGATGCCTCAACTCTCCAG |
| Snail family transcriptional repressor 1 | SNAI1 | CGAGTGGTTCTTCTGCGCTA | GGGCTGCTGGAAGGTAAACT |
| Snail family transcriptional repressor 2 | SNAI2 | GCTACCCAATGGCCTCTCTC | CTTCAATGGCATGGGGGTCT |
| Mesoderm posterior BHLH transcription factor 1 | MESP1 | GACCCATCGTTCCTGTAC | CTGAAGAGCGGAGATGAG |
| Paired box 6 | PAX6 | GAGCGAGCGGTGCATTTG | TCAGATTCCTATGCTGATTGGTGAT |
| Nestin | NES | CCACCCTGCAAAGGGAATCT | GGTGAGCTTGGGCACAAAAG |
| E-cadherin | CDH1 | GCTGGACCGAGAGAGTTTCC | CAAAATCCAAGCCCGTGGTG |
| N-cadherin | CDH2 | CATTGCCATCCTGCTCTGCATC | GCGTTCTTTATCCCGGCGTTTC |
| P-cadherin | CDH3 | GACACCCATGTACCGTCCTC | TTCTGCGGCAACAGAGAACA |
| Lymphoid enhancer binding factor 1 | LEF1 | CCCGTGAAGAGCAGGCTAAA | AGGCAGCTGTCATTCTTGGA |
| Beta catenin | CTNNB1 | GCGCCATTTTAAGCCTCTCG | GAGTAGCCATTGTCCACGCT |
| Junction plakoglobin | CTNNG | CCCCATACTCAGTAGCCACG | CATCCTCCTCCATGATGCCC |
| Alpha catenin | CTNNA1 | TCGGGCCTCTGGAATTTAGC | CAGCCAAAACATGGGCCTTC |
| Protocadherin 8 | PCDH8 | GCTGATCGTCATCATCGTGC | AAGGTGAGCACGTCGAACAT |
| Protocadherin 1 | PCDH1 | GGGACTGACTGCTCTTGTGG | CTCCCAATGAGGGTGTTGGG |
| Gap junction protein gamma 1 | GJC1 | CCCGTGCTACAATGGACCAA | TCTAGCAGGCGAGTCAGGAA |
| Gap junction protein alpha 1 | GJA1 | AGCCACTAGCCATTGTGGAC | CCACCTCCACCGGATCAAAA |
| Integrin subunit alpha v | ITGAv | ACAAATGCTCCTAGGCACCC | GCGGGTAGAAGACCAGTCAC |
| Integrin subunit alpha 4 | ITGA4 | CAGGTTTAAAGCATGGCCACA | TGGCATTGGCATTGTGTACC |
| Integrin subunit beta 3 | ITGB3 | ACCAGTAACCTGCGGATTGG | TCCGTGACACACTCTGCTTC |
| Integrin subunit beta 1 | ITGB1 | GCCGCGCGGAAAAGATG | ACATCGTGCAGAAGTAGGCA |
| Integrin subunit alpha 1 | ITGA1 | GGCAGCACAATTCATGCACA | AAATGTACACAGCTCCCCCG |
| Integrin subunit alpha 3 | ITGA3 | TAGGCAGCTTCATCCTGCAC | GTCCTGCCACCCATCATTGT |
| Integrin subunit alpha 5 | ITGA5 | TTACGGGACTCAACTGCACC | AGCCTGAAACACTCAGCCTC |
| Integrin subunit alpha 6 | ITGA6 | ACACAGCATTGTATATGTGAAGCA | CCGAATCCCATTGCTTTGGC |
| Integrin subunit beta 2 | ITGB2 | CAACCCACCACTTCCTCCAAG | ACTTCGTGCACTCCTGAGAGA |
| Integrin subunit alpha 2 | ITGA2 | CGGTTATTCAGGCTCACCGA | CCTCGGGGCCTTCAAGAAAT |
| Epithelial cell adhesion molecule | EPCAM | GAACACTGCTGGGGTCAGAA | TCCTTCTGAAGTGCAGTCCG |
| Rho associated coiled-coil containing protein kinase 1 | ROCK1 | GTTTGAACAGGAAGGCGGAC | ATGCCCGATGGAGACTTAGC |
| Rho associated coiled-coil containing protein kinase 2 | ROCK2 | TCCCGATAACCACCCCTCTT | TGCCTTCATCTGTAGACCTCTG |
| Myosin heavy chain 9 | MYH9 | TGGTTTACCTGCACCGTTGA | TTCGGCAACCAGTGTAGACC |
| Myosin heavy chain 10 | MYH10 | GGTGGGTTTGGGACTGAGG | ACAGCCCTGTCCACAAAGAG |
| Myosin light chain 9 | MYL9 | TTGACAAGGAGGACCTGCAC | GCGTTGCGAATCACATCCTC |
| Myosin light chain 12B | MYL12B | CCCTGTGCCCAACACTATCC | ACACATTGGATGTTGCACGC |
| Myosin light chain 12A | MYL12A | GCACTTGGTCAATACCACGC | CGAGAATCCGAGCACCTCTC |
| Ras homolog family member A | RHOA | ACACACCAGGCGCTAATTCA | CCCCAGAGCTATGCCAACAA |
| Protein tyrosine kinase 2 | PTK2 | TGGGCGGAAAGAAATCCTGC | GCCCGTCACATTCTCGTACA |
| Mitogen-activated protein kinase 8 | MAPK8 | CTGAAGCAGAAGCTCCACCA | CACCTAAAGGAGAGGGCTGC |
| EPH receptor A2 | EPHA2 | TTCCATTAAGGACTCGGGGC | ATCAGGTCCCACTTCCTTGC |
| EPH receptor A3 | EPHA3 | GCTGAAGACGGCACTAGGAC | AGGGCAGTGAGAGGAGCATA |
| EPH receptor A4 | EPHA4 | ACGCGTGCTCATCTTGTGTA | GTCGAGGAGAGGACAGGTCT |
| EPH receptor B2 | EPHB2 | CAGCATTACCCTGTCGTGGT | GGGGTCACTTCTGTCATGGT |
| EPH receptor B4 | EPHB4 | GTCCCGCGCGGAGTATC | CCTGAGGGAATGTCACCCAC |
| Ephrin A5 | EFNA5 | CTTCTCGCTCTCCTACCCCT | ACACACATCCAGAGCACCAG |
| Ephrin B2 | EFNB2 | GGAAGTACTGCTGGGGTGTT | GTGCATCTGTCTGCTTGGTCT |
| Beta-actin | ACTB | ACAGAGCCTCGCCTTTGCC | GAGGATGCCTCTCTTGCTCTG |
| Death associated protein kinase 3 | DAPK3 | GAGAATCTGAGGAGCTGGGTT | GAACTTGGCTGCGTACTCCT |
